# The Effects of Surgical Androgen Deprivation Therapy for Advanced Prostate Cancer on Peripapillary Retinal Nerve Fiber Layer Thickness

**DOI:** 10.5152/eurasianjmed.2025.24597

**Published:** 2025-05-12

**Authors:** Feyzahan Uzun, Hüseyin Fındık, Muhammet Kaim

**Affiliations:** Department of Ophthalmology, Recep Tayyip Erdoğan University School of Medicine, Rize, Türkiye

**Keywords:** Androgen deprivation therapy,, optic coherence tomography,, orchiectomy,, retinal nerve fiber layer thickness,, testosterone

## Abstract

**Background:**

Prostate cancer is the most common malignancy in men, and androgen deprivation therapy (ADT) serves as the primary approach for managing advanced cases. Certain research has suggested the impact of androgens on the physiological homeostasis of the optic nerve. Our aim was to investigate the impact of surgical ADT on peripapillary retinal nerve fiber layer (RNFL) thickness in patients with advanced prostate cancer.

**Methods:**

The study comprised 30 patients who had undergone bilateral orchiectomy for advanced prostate cancer, with a total of 60 eyes included in the analysis. Each participant received a standard ophthalmological examination. Peripapillary RNFL thickness measurements were performed preoperatively and 12 months postoperatively using optical coherence tomography.

**Results:**

The mean age of the patients was 73.77 ± 8.8 years. At the 12th month following surgery, we observed that the mean average thickness of the right and left RNFL, as well as the thickness of the left nasal, left inferior, and right and left temporal quadrants, was significantly thinner compared to presurgical values (*P* < .05). However, when comparing pre- and postsurgical measurements, the RNFL thickness in the right and left superior quadrants, as well as the right nasal and right inferior quadrants, showed no statistically significant difference.

**Conclusion:**

In this study, a significant difference was observed between the preoperative and 12-months postoperative peripapillary RNFL thickness values in patients who underwent surgical ADT for advanced prostate cancer. Additional research using larger sample sizes is required to clinically examine the impact of ADT on optic nerve homeostasis.

**Main Points**

- Androgen deprivation therapy (ADT) is the main treatment strategy for managing locally recurrent or advanced cases of prostate cancer.
- Bilateral orchiectomy, one of the methods of ADT, rapidly suppresses serum testosterone levels.
- Previous studies have shown that sex hormones have an effect on the optic nerve.
- In this study, we aimed to investigate the impact of surgical castration on peripapillary retinal nerve fiber layer thickness in patients with advanced prostate cancer.

## Introduction

Prostate cancer is the most prevalent malignancy in men, and androgen deprivation therapy (ADT) is the main treatment strategy for managing locally recurrent or advanced cases of the disease.^1^ Androgen deprivation can be accomplished through either surgical means, such as bilateral orchiectomy or hormone-based medical interventions including luteinizing hormone-releasing hormone (LHRH) analogs/antagonists, anti-androgens, or estrogens.^1^ Bilateral orchiectomy, which involves the complete removal of the testes, effectively and rapidly suppresses serum testosterone within 24 hours with a one-time cost procedure and is associated with lower risks of cardiovascular complications compared to hormonal therapy.^2,3^

Sex hormone receptors have been identified in several eye tissues, including the cornea, conjunctiva, lacrimal and meibomian glands, iris, lens, as well as retinochoroidal structures.^4^ A recent systematic review indicated that female sex hormones influence the ocular surface, cornea, and intraocular pressure (IOP) through direct receptor interactions, as well as indirectly through immune and vascular-mediated processes.^5^ Although the mechanism is not entirely clear, certain research has suggested the impact of sex hormones including estrogen, androgen, and progesterone on the physiological homeostasis of the optic nerve.^6^ Estrogens and progesterone have demonstrated antioxidant, anti-inflammatory, and neuroprotective effects. Researchers have claimed that low-dose estrogen therapy may exert a protective effect against glaucoma by stimulating collagen fiber synthesis around the lamina cribrosa, which reduces compression on retinal ganglion cell axons.^7^ Androgens play a physiological role in the central nervous system,^8^ and androgen deprivation therapy has been linked to decreased neuronal activity and cognitive function in men.^9^ Meanwhile, testosterone, by acting on neural androgen receptors, promotes myelin regeneration and provides trophic and neuroprotective benefits to the optic nerve.^6^ It has been suggested that estrogen enhances ocular blood flow through vasodilation, whereas testosterone appears to exhibit antagonistic effects compared to estrogen regarding ocular blood flow.^10^ Age, sex, and physiological hormonal fluctuations such as those occurring during the menstrual cycle, pregnancy, menopause, and andropause may influence the distribution and configuration of gonadal hormone receptors in ocular tissues, leading to variations in ocular physiopathology.^4^ Thus these factors have been associated with different and conflicting results in the literature.

Measurements of retinal nerve fiber layer (RNFL) thickness offer crucial clinical insights into disorders of the posterior ocular structures. Advances in optical coherence tomography (OCT) technology now allow for high-quality imaging of the neuroretina. Most studies investigating the influence of testosterone on RNFL thickness have included women with supraphysiological androgen levels, such as those in postmenopause,^11^ polycystic ovarian syndrome (PCOS),^12^ and those who use exogenous testosterone for transgenderism.^13^ Due to the relatively stable testosterone levels in males, it has been challenging to assess the impact of testosterone on the risk factors and pathophysiological processes of the optic nerve. With ADT, serum testosterone rapidly and abruptly decreases to non-physiological levels. Therefore, data demonstrating the impact of testosterone on the optic nerve is highly valuable in this group of patients. The association of medical ADT with glaucoma has been investigated by some researchers in large population-based cohorts recently^14^ but to the best of our knowledge, no earlier studies have been conducted to assess the role of sudden and rapid decreases in testosterone levels through bilateral orchiectomy on the optic nerve. In the current study, our aim was to investigate the impact of surgical castration on peripapillary RNFL thickness in patients with advanced prostate cancer over a 12-months period.

## Material and Methods

This study was conducted at a tertiary university hospital, and Recep Tayyip Erdoğan University Ethics Committee (Approval No.: 2018/90, Date: 16.09.2018) approval was obtained prior to the study. Researchers involved in the study adhered to the principles of the Declaration of Helsinki, and all participants provided written consent for data collection after receiving an explanation about the study’s purpose and procedures.

**Figure 1.**
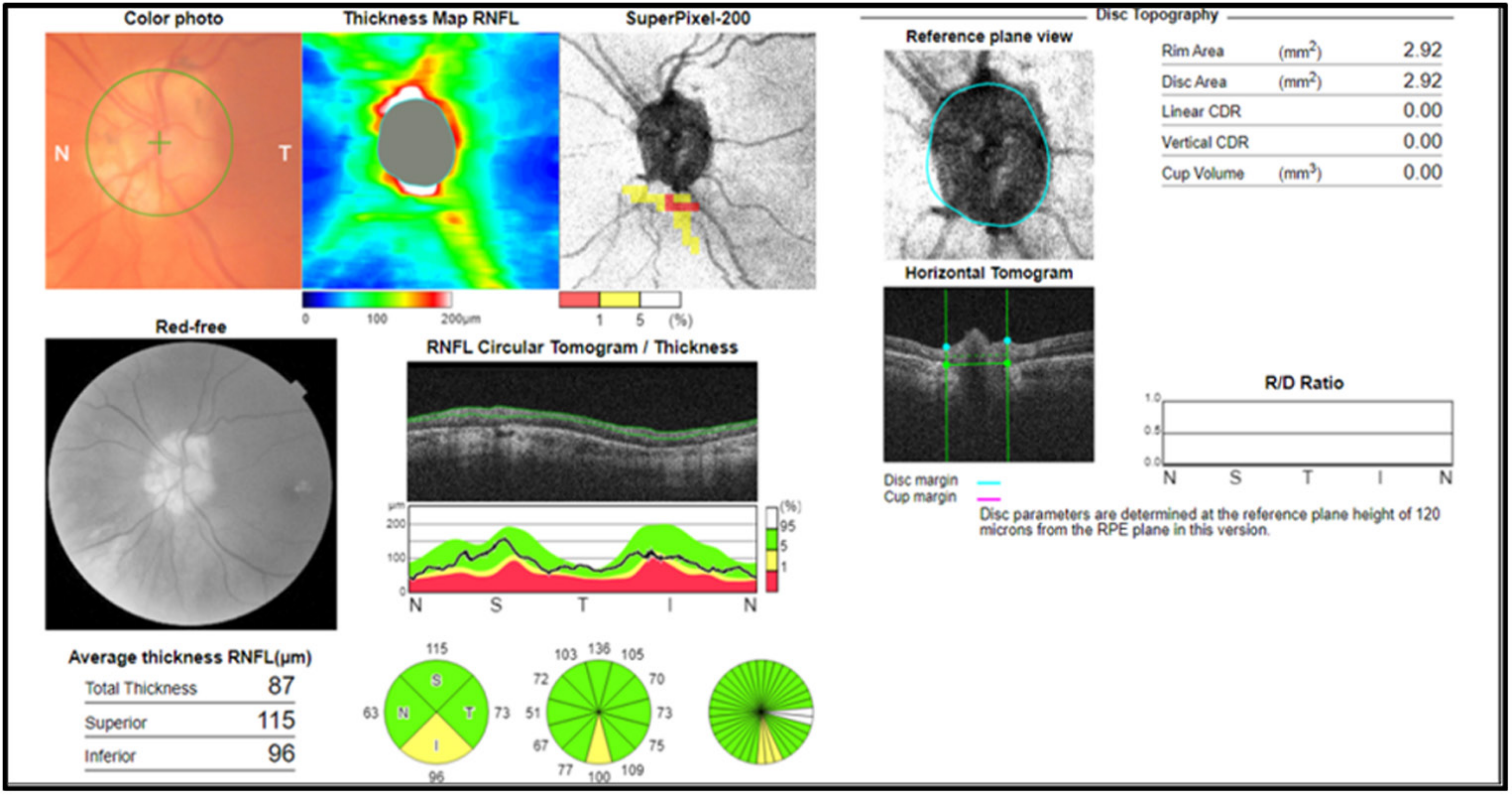
Measurement of retinal nerve fiber layer (RNFL) thicknesses with OCT. OCT, optical coherence tomography.

The study included 60 eyes of 30 patients who underwent bilateral orchiectomy for advanced prostate cancer between January 2018 and December 2022. All patients underwent a comprehensive ophthalmologic examination including best-corrected visual acuity, slit lamp examination, Goldmann applanation tonometer, and fundoscopy preoperatively at baseline as well as at the 12th month postoperatively. Patients with preexisting glaucoma, any neurologic or systemic disease that may affect the optic nerve, a history of retinal vascular disease, laser procedures, retinal pathology, retinal surgery, smoking habits, or refractive errors exceeding 2 diopters were excluded from the study. The peripapillary RNFL thickness images were acquired by the same technician using the swept-source OCT (DRI-OCT-1, Topcon, Tokyo, Japan). A standard protocol that uses a 360-degree circular scan with a diameter of 3.4 mm around the optic disc was employed to investigate the temporal, nasal, inferior and superior quadrants, and the average thickness of the RNFL (Figure 1). Poor-quality OCT images (quality score <45) were not included in the study.

### Statistical Analysis

Data analysis was conducted using SPSS for Windows, version 23 (IBM SPSS Corp.; Armonk, NY, USA). The Kolmogorov–Smirnov test was used to assess the normality of the distribution of continuous variables, and this test indicated a normal distribution for all groups (*P* > .05). Continuous variables were presented as mean ± standard deviation (SD). The mean difference between preoperative and postoperative values was compared using a paired *t*-test. A *P*-value of less than .05 was considered statistically significant.

## Results

The mean age of the patients was 73.77 ± 8.8, ranging from 66 to 95 years. Demographic characteristics of all patients are shown in Table 1. Any retinal problem or vision loss were observed in none of the patients during the study period. The mean preoperative average RNFL thickness of the right and left eyes was 101 ± 13.52 μm and 99 ± 11.38 μm, respectively. The mean postoperative average RNFL thickness of the right and left eyes was 94 ± 15.33 μm and 91 ± 19.73 μm, respectively. Quadrantal peripapillary RNFL thickness (superior, nasal, inferior, and temporal) measurements of both eyes in the pre- and postoperative periods are shown in Table 2. At the 12th month following ADT surgery, we observed that the average thickness of the right and left RNFL, as well as the thickness of the left nasal, left inferior, and right and left temporal quadrants, was significantly thinner compared to presurgical values (*P* < .05). However, when comparing pre- and postsurgical measurements, the RNFL thickness in the right and left superior quadrants, as well as the right nasal and right inferior quadrants, showed no statistically significant difference (*P* > .05).

## Discussion

In the current study, patients with advanced prostate cancer who underwent surgical orchiectomy showed a reduced average RNFL thickness. Specifically, the RNFL exhibited significant thinning in the nasal, inferior, and temporal quadrants during the postoperative period compared to the preoperative values.

Specific androgen hormone receptors have been discovered in numerous ocular tissues, and androgens have been linked to the development of several eye diseases, including dry eye disease, meibomian gland disorders, and glaucoma.^15^ Testosterone plays an important role in neurophysiological processes in the central nervous system.^16^ While the impact of testosterone on the optic nerve, as an extension of the brain, remains unclear, previous studies have suggested that testosterone may have trophic and neuroprotective effects, as well as help regulate ocular blood flow.^6,10^ In men, due to the relatively stable levels of testosterone that do not significantly deviate from physiological ranges, research regarding the impact of non-physiological androgen levels on the optic nerve has predominantly focused on women. de Souza-Júnior et al observed a significantly thicker RNFL in the superior quadrant of the optic nerve in individuals with PCOS compared to healthy subjects.^17^ Shiromani et al observed a similar thickening of the RNFL in the superior quadrant among PCOS patients, particularly those with a body mass index exceeding 30 kg/m^2^.^12^ Additionally, Alpogan et al^13^ found that the mean RNFL thickness in the PCOS group exceeded that of the healthy female cohort in all quadrants, although the difference was not statistically significant. The postmenopausal phase and the administration of exogenous testosterone for transgender individuals represent additional states characterized by supraphysiological levels of androgens in women. Fathy et al^18^ reported that hormonal shifts occurring postmenopause lead to decreases in both RNFL thickness and optic nerve perfusion. Alpogan et al observed no difference in RNFL and ganglion cell complex (GCC) measurements between premenopausal women and those in the postmenopausal stage. However, they noted that an extended postmenopausal duration correlated with elevated RNFL and GCC values. The researchers postulated that increasing ovarian testosterone secretion after menopause may have a neuroprotective and neuroregenerative effect on the optic nerve, thereby protecting the retinal nerve layer from the effects of aging and menopause.^11^ An investigation revealed that the utilization of supraphysiological doses of testosterone resulted in elevated IOP and augmented thicknesses of the macula and RNFL in female-to-male transgender persons. In that study, the authors concluded that the increase in RNFL was attributed to the trophic and neuroprotective effects of testosterone on the optic nerve.^19^

Androgen deficiency occurs in men during the physiological aging process, in cases of complete androgen insensitivity syndrome, or in individuals receiving anti-androgen therapy—either surgical or medical. The relationship between androgen deficiency and dry eye disease, along with meibomian gland dysfunction, has been extensively investigated to date.^20^ Despite some studies investigating the association between androgens and glaucoma, a clear understanding has not yet emerged, and there remain contradictory results on this matter. Bailey et al^21^ reported that a group of single nucleotide polymorphisms in genes involved in testosterone metabolism was linked to primary open-angle glaucoma (POAG) in men, though the specific genes responsible for these findings lacked consistent identification. Researchers conducted a retrospective nationwide cohort study using a database and found that medical ADT was linked to a reduced risk of POAG in Korean patients with prostate cancer.^22^ The same research group indicated a trend toward reduced prevalence of normal-tension glaucoma (NTG) in patients undergoing medical ADT for prostate cancer in another study.^14^ They speculated that testosterone might play a role in the pathogenesis of NTG, and castration-levels of testosterone could potentially offer benefits in regulating IOP and ocular blood flow. However, the last 2 studies have several limitations. These include the absence of data on systemic medications known to affect glaucoma, insufficient information on serum testosterone levels, inadequate details regarding IOP, and the lack of results from optic nerve examinations in their database. On the other hand, Sonmez et al^23^studied alterations in extraocular muscle mass, retroorbital fat (ROF), and the optic nerve in patients who underwent ADT exclusively with LHRH agonists for a minimum of 12 months. This radiological study, which evaluated computed tomography scans, revealed thinning of the extraocular muscles, increased ROF mass, and elongation and thickening of the optic nerve. The authors speculated that the changes in the optic nerve may be associated with the change in ROF and mechanical ocular protrusion. It has been demonstrated that the optic nerve contains LHRH fibers^24^ and receptors.^25^ Therefore, when achieving ADT with LHRH analogs, it is imperative to consider the additional impact of LHRH on the optic nerve. Moreover, LHRH analogs may induce papilledema and pseudotumor cerebri as potential side effects.^26^ In our study, we observed a mean average and quadrantal RNFL thinning 12 months following bilateral orchiectomy.

Our study has several limitations, such as a relatively small sample size and the lack of a control group to evaluate the impact of the natural progression of prostate cancer on the optic nerve. The follow-up period of our study was relatively limited, as the mean survival of patients with metastatic prostate cancer is between 5 and 10 years.^27^ However, the strengths of this study lie in its prospective nature and the fact that ADT was achieved through surgery, thus eliminating the consideration of side effects from hormonal medications. Consequently, our research holds particular significance for elucidating the direct impact of testosterone deficiency on the optic nerve.

In conclusion, androgens appear to play a role in the pathophysiological mechanisms of the optic nerve, as evidenced by our findings suggesting a potential relationship between androgens and peripapillary RNFL thickness. However, additional experimental and clinical studies with larger sample sizes and longer follow-up periods are needed to fully understand the impact of androgens on the optic nerve.

## Figures and Tables

**Table 1.** Demographic Characteristics of the Patients

	N = 30	
Age (years) (mean ± SD)	73.77 ± 8.8 (range 59-88)	
Baseline BCVA (Snellen)	0.89 ± 0.04	
	Preoperative	Postoperative
IOP (mm Hg)	14.68 ± 1.4	13.23 ± 1.9
Serum PSA (ng/mL)	45.2 ± 2.5 (range 20- >100)	3.12± 0.8 (range 1.7-7.4)
Serum testosterone (ng/dL)	358.3 ± 2.3 (range 155.1-632.3)	11.9 ± 1.4 (range 8.3-17)

BCVA, best-corrected visual acuity; IOP, intraocular pressure; PSA, prostate-specific antigen.

**Table 2.** The Comparison of Average and Quadrantal Retinal Nerve Fiber Layer Thickness Between Preoperative and Postoperative Measurements

	Preoperative	Postoperative	*P*
Average RNFLT			
Right	101 ± 13.52	94 ± 15.33	**.03**
Left	99 ± 11.38	91 ± 19.73	**.04**
Superior			
Right	115.03 ± 21.79	113.10 ± 20.66	.49
Left	113.45 ± 22.85	111.97 ± 32.89	.25
Nasal			
Right	73.03 ± 13.65	69.52 ± 10.52	.08
Left	75.01 ± 11.21	67 ± 15.2	**.02**
Inferior			
Right	127.26 ± 19.27	121.82 ± 22.01	.62
Left	129 ± 12.1	119 ± 14.3	**.018**
Temporal			
Right	79 ± 11.5	73 ± 17.2	**.01**
Left	76 ± 13.7	70 ± 18.1	**.01**

**Bold**: statistically significant.

RNFLT, retinal nerve fiber layer thickness.

## Data Availability

The data that support the findings of this study are available on request from the corresponding author.
